# Management of Anterior Choroidal Artery Aneurysms: A Retrospective Cohort Study

**DOI:** 10.3390/brainsci15010005

**Published:** 2024-12-24

**Authors:** Andrew Falzon, Shigeta Miyake, Tze Phei Kee, Hugo Andrade-Barazarte, Timo Krings

**Affiliations:** 1 Department of Neuroradiology, Atkinson Morley Regional Neuroscience Centre, St George’s University Hospital, Tooting, London SW17 0QT, UK; 2 Department of Neurosurgery, Yokohama City University School of Medicine, Yokohama 236-004, Japan; 3 Department of Neuroradiology, National Neuroscience Institute, Singapore 308433, Singapore; 4 Division of Neurosurgery, Toronto Western Hospital, Toronto, ON M5T 2S8, Canada; 5 Sprott Department of Surgery, University of Toronto, Toronto, ON M5T 2S8, Canada; 6 Division of Neurointerventional Radiology, UMass-Chan Lahey Department of Radiology, Lahey Hospital and Medical Centre—Beth Israel Lahey Health, Burlington, MA 01805, USA; 7 TH Chan School of Medicine, UMass Chan Medical School, Worcester, MA 01655, USA

**Keywords:** anterior choroidal artery aneurysm, endovascular, flow diverter stent, balloon-assisted coiling, stent-assisted coiling, clipping

## Abstract

**Background:** Anterior choroidal artery (AChoA) aneurysms pose a challenge for both endovascular and clipping procedures. The eloquent territory supplied by the parent vessel has limited collateralization and its compromise can lead to significant morbidity. This study aims to analyze the clinical outcomes and procedure-related complications of clipping and endovascular treatment of AChoA aneurysms to aid physician decision making. **Methods:** Thirty-two ruptured and unruptured AChoA aneurysms that underwent catheter angiography at a single neurovascular center between January 2000 and December 2023 were included. Either conservative management, clipping, and/or endovascular treatment were performed. Clinical outcomes and complications were analyzed retrospectively. **Results:** Twenty-four endovascular treatments and seven clipping procedures were included. Of the total 24 endovascular procedures, 46% were primary coiling, 25% were balloon-assisted coiling, 13% were flow diverting stent, 8% were combined balloon-assisted coiling and flow diverting stent, and 8% were combined balloon-assisted and stent-assisted coiling. There was no procedure-related mortality in both groups. No intra- or post-procedural ruptures/re-ruptures occurred during follow-up in both endovascular and clipping cohorts. AChoA territory infarcts occurred in 4% of the endovascular and 29% of the clipping cohorts. Other thromboembolic complications occurred in 4% of the endovascular cohort. The recurrence rate requiring retreatment was 12.5% for the endovascular and 43% for the clipping cohort. A favorable clinical outcome (mRS ≤ 2) was 78% for the endovascular cohort and 67% for the clipping cohort. **Conclusions:** Endovascular and clipping outcomes align with prior studies, with endovascular showing a favorable safety profile. Both approaches are viable, though they present distinct risks and advantages.

## 1. Introduction

Anterior choroidal artery (AChoA) aneurysms account for a small proportion of all intracranial aneurysms, comprising 2–5% of the total [1]. The treatment of these aneurysms may pose challenges due to their complex anatomical and hemodynamic characteristics, which are prone to ischemic stroke [2]. Both endovascular and surgical treatment options are possible, each with distinct advantages and disadvantages [3].

More recent advances in endovascular technology and procedural techniques allowed for many cases to be effectively treated this way [4,5]. The efficacy and safety of these two treatment modalities were described previously in relatively small case series due to the low incidence of these aneurysms. A recent meta-analysis of these case series has provided further insight into this rare entity and its treatment; however, many limitations and unknowns remain.

The purpose of this study is to analyze the clinical outcomes and procedure-related complications of endovascular treatment and clipping of the AChoA aneurysms in a single tertiary hospital to help aid future management and improve patient outcome.

## 2. Materials and Methods

This retrospective cohort study reviewed ruptured and unruptured AChoA aneurysms that underwent assessment with catheter angiography and subsequent conservative management, clipping, or endovascular treatment at a single neurovascular tertiary center between January 2000 and December 2023.

A total of 32 patients were recruited. Nineteen patients underwent primary endovascular treatment. Of these, two patients required endovascular retreatment and one patient required retreatment with clipping. Six patients underwent primary clipping. Of these, three patients required endovascular retreatment. A total of 24 endovascular and 7 clipping procedures were performed.

Twelve patients were initially managed conservatively with modification of risk factors and regular imaging surveillance. Of these, four patients underwent subsequent primary endovascular treatment and one underwent primary clipping. Treatment was not indicated in the remaining seven patients.

The inclusion criteria were: patients with an unruptured or ruptured AChoA aneurysms and (1) pre-treatment digital subtraction angiography; (2) rotational angiography with 3D reconstruction; (3) accessible electronic medical records; and (4) underwent clipping, endovascular treatment, and/or conservative management.

The exclusion criteria were: (1) atypical aneurysms, including dissecting, blister-type, or fusiform; and (2) patients lacking sufficient Digital Imaging and Communications in Medicine (DICOM) data for accurate volume rendering. This ensured more accurate aneurysm characterization.

All clinical data were obtained from a local hospital research database and individual electronic patient records. Hypertension was defined as at least two separate blood pressure measurements recorded in medical records with a systolic blood pressure > 140 mm Hg or a diastolic blood pressure of >90 mm Hg. Smoking was defined as current cigarette smoking at the time of admission with cumulative smoking history of at least five pack years. Family history of aneurysm was defined as having two first degree relatives with a diagnosed intracranial aneurysm.

Data were analyzed using IBM SPSS Statistics 29.0.2.0 for Mac. Continuous variables were demonstrated as mean/median and range. Categorical variables were demonstrated as total numbers and percentages. A STROBE checklist was utilized as a guide throughout.

### 2.1. Endovascular Procedural Details

All sterile procedures were performed under general anesthesia using a closed system of continuous heparinized saline flush. In total, 22/24 (92%) were performed using right common femoral artery access and 2/24 (8%) were performed using right radial artery access.

Systemic heparinization was administered intravenously with an initial bolus of 100 IU per kg of body weight, and 1000 IU administered every hour after. If radial access was used, a cocktail containing 3000 IU of heparin, 2.5 mg of verapamil, and 150 mcg of nitroglycerin was administered intra-arterially, with additional IV heparin top up. Diagnostic angiography, including rotational angiography with 3D volume rendering, was performed and analyzed to determine the most appropriate endovascular or surgical approach. Endovascular approaches included primary coiling, balloon assisted coiling (BAC), stent assisted coiling (SAC), and flow diverting stent (FDS).

All elective patients were loaded on dual antiplatelet therapy for five days prior to the procedure with Aspirin 81 mg PO OD and Ticagrelor 90 mg PO BD or Clopidogrel 75 mg OD. The choice of antiplatelet therapy was based on operator preference. No platelet function testing was performed. If a flow divertor was implanted, dual antiplatelet therapy was continued for three months. This was converted to single antiplatelet therapy with Aspirin only if follow up surveillance imaging was satisfactory. All acute patients were treated with primary coiling and were not loaded on antiplatelet therapy.

### 2.2. Clipping Procedural Details

In our approach, the treatment modality would be determined after 3D angiographic evaluation. Endovascular approach would be considered unless an unfavorable outcome was anticipated, therefore requiring clipping.

Three cases were performed through an ipsilateral lateral supra-orbital craniotomy and four cases through an ipsilateral pterional craniotomy. One of these cases was referred with limited intra-operative information. A sub-frontal approach was performed using microsurgical techniques. In all six cases, patency of the AChoA was verified intra-operatively using indocyanine green (ICG) video angiography. Somatosensory and motor evoked potentials (SSEPs and MEPs) were also used to monitor neurological dysfunction intra-operatively.

### 2.3. Multidisciplinary Approach and Neurovascular Experience

The treatment modality would be determined following angiographic assessment and discussion within a neurovascular board meeting or mini multidisciplinary meeting.

The neurovascular group consists of senior operators, including microvascular neurosurgeons, interventional neuroradiologists, and hybrids. An endovascular approach would typically be considered the preferred first line treatment unless an unfavourable outcome was anticipated, therefore requiring clipping

The institution is a high-volume teaching hospital with approximately 50–65 patients seen in a multidisciplinary neurovascular clinic each week. This includes new referrals and follow-up for microvascular or endovascular procedures. Lead operators include a microvascular neurosurgeon with 15 years out of fellowship and interventional neuroradiologists with 5–20 years out of fellowship. Approximately 70% of treated aneurysms are via endovascular approach, with the remaining being surgical. Exact procedural numbers vary from year to year.

### 2.4. Post-Procedural Follow-Up

Routine CT or MR angiography follow-up was performed in accordance with local guidelines for all aneurysm patients in our center. Pre- and post-procedural clinical assessments were performed by an interventional neuroradiologist and/or a vascular neurosurgeon. The clinical follow-up was performed through our multidisciplinary outpatient neurovascular clinic. If the patient was lost to follow-up or repatriated to their local institution, the latest clinical outcome available was used.

### 2.5. Evaluation of Treatment Efficacy, Clinical Outcome, and Complications

All imaging data were reviewed by an interventional neuroradiologist. Treatment efficacy was evaluated using immediate post-procedural modified Raymond Roy classification (mRRC) and by reviewing follow-up imaging data for recurrence, residuum growth, and re-rupture.

Clinical outcome for all patients was evaluated using the modified Rankin scale (mRS) before and after intervention at the last available follow-up. A favorable mRS was defined as ≤2. A poor outcome was defined as >2.

For the ruptured cases, the World Federation of Neurological Surgeons grading scale (WFNS) and Hunter and Hess (HH) scale were obtained from medical records. Modified fisher grade was evaluated on the initial non-contrast CT head and interpreted by a blinded neuroradiologist.

Intra-procedural complications were evaluated by reviewing angiographic images and procedure notes. Post-procedural complications were evaluated by reviewing clinic notes and surveillance imaging. The incidence of vasospasm secondary to subarachnoid hemorrhage (SAH) was analyzed as a factor impacting clinical outcome.

## 3. Results

For the total 32 patients, the mean age was 50, with a range of 20–80 years. There were 5/32 (16%) males and 27/32 (84%) females; 5/32 (16%) patients had hypertension, 14/32 (44%) patients had multiple intracranial aneurysms, and 4/32 (13%) had previous SAH from another aneurysm rupture.

There were 17/32 (53%) ruptured AChoA aneurysms, and 15/32 (47%) were unruptured. For the ruptured aneurysms, the mean PHASES score was 4.5 with a range of 4–7. For the unruptured aneurysms, the mean PHASES score was 4.9 with a range of 4–10 (Table 1).

Aneurysm size for the entire cohort had a mean of 3.9 mm, with a range of 1.3–11 mm. For the ruptured cohort, the mean size was 4.2 mm with a range of 1.3 to 7 mm. For the unruptured cohort, the mean size was 3.5 mm with a range of 2 to 11 mm.

Aneurysm neck diameter had a mean of 2.4 mm with a range of 1.1–5 mm. For the ruptured patients, the mean neck was 2.6 mm with a range of 1.5 to 3.6 mm, and for the unruptured patients, the mean neck was 2.2 mm with a range of 1.1 to 3.6 mm.

Aspects ratio had a mean of 1.74, with a range of 0.87–3.44. For the ruptured cases, the mean was 1.74 with a range of 0.87 to 3.16 mm. For the unruptured cases, it was 1.60 with a range of 1 to 3.44.

### 3.1. Conservative Management

Conservative management was indicated in seven patients during the entirety of their follow-up. These aneurysms did not demonstrate an appreciable change in volume or morphology over a median of 31 months of follow-up with an interquartile range of 50.5 months. The average aneurysm size of this group was 2.4 mm, with a median of 2.3 mm and a range of 2–2.6 mm.

### 3.2. Endovascular and Clipping Cohorts

A total of 25/32 (78%) patients were treated; 12/32 (38%) were initially managed conservatively. Of these, 5/12 (42%) required treatment and 7/12 (58%) did not.

**Figure 1:**
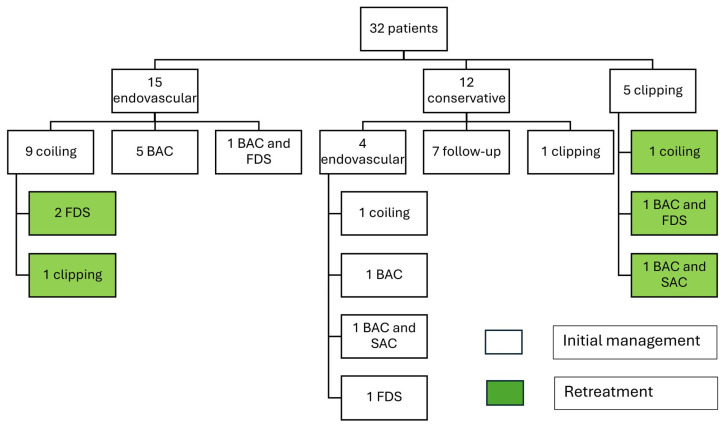
Schematic representation of the conservative, endovascular, and clipping management of each patient. Retreatments are highlighted in green.

A total of 24 endovascular treatments were performed; 19/24 (79%) were primary endovascular cases, 2/24 (8%) were secondary treatments following primary endovascular treatment, and 3/24 (13%) were secondary treatments following primary clipping (Figure 1).

Of all 19 primary endovascular cases, 10/19 cases (53%) underwent primary coiling, as a part of the initial management, 6/19 (32%) underwent BAC alone, 1/19 (5%) underwent combined BAC and SAC, 1 case (5%) underwent combined BAC and FDS, and 1 case (5%) underwent FDS.

Of all 5 secondary endovascular cases, 2/5 (40%) were initially treated with primary coiling and retreated with FDS, while 3/5 (60%) were initially treated with primary clipping and retreated with various endovascular techniques, including 1/5 (20%) BAC and FDS, 1/5 (20%) BAC and SAC, and 1/5 (20%) coiling alone.

A total of 7 clipping treatments were performed. Of these, 6/7 (86%) were primary clipping cases and 1/7 (14%) was secondary treatment following primary endovascular treatment.

### 3.3. Ruptured and Unruptured Cohorts

A total of 21/31 (68%) procedures were for the ruptured aneurysms and 10/31 (32%) were for the unruptured aneurysms. Within the endovascular cohort, 13/24 (54%) patients had a ruptured aneurysm and 11/24 (46%) patients had an unruptured aneurysm. Within the clipping cohort, 5/7 (71%) patients had a ruptured aneurysm and 2/7 (29%) patients had an unruptured aneurysm.

### 3.4. Procedure Related Complications

The complications of the 24 endovascular cases and 7 clipping cases are discussed below.

#### 3.4.1. Procedure Related Infarction

A total of 4/31 (13%) patients experienced thromboembolic complications, 2 in the endovascular cohort (2/24 = 8.3%) and 2 in the surgical cohort (2/7 = 28.5%). All four patients were ruptured pre-procedure (Table 2).

Within the endovascular cohort, one patient had an AChoA territory infarct (Figure 1) and the other had a catheter-related PCA (posterior cerebral artery) and MCA (middle cerebral artery) territory infarct. The catheter-related infarct presented with limb hemiparesis after 4 h, which resolved after 1 week.

**Figure 2:**
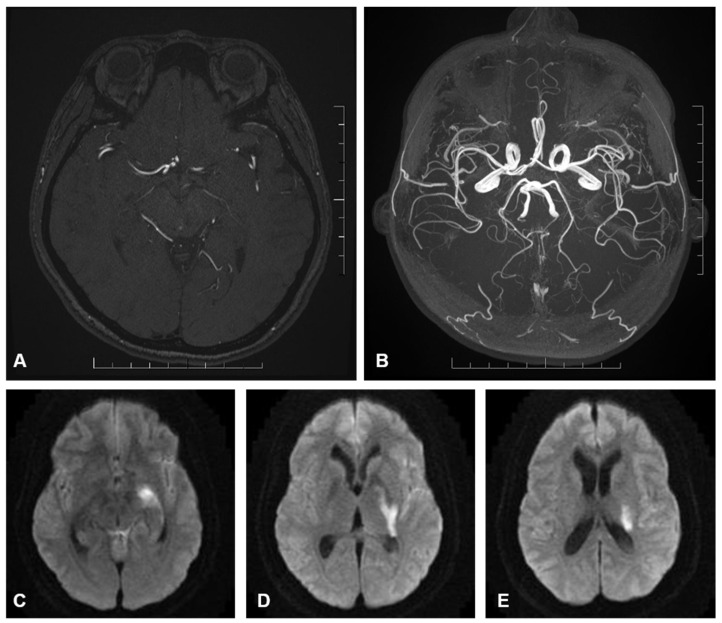
(**A**) axial image of a contrast enhanced MR intracranial angiogram which demonstrates the course of the AChoA. The artery gives rise to multiple perforators as it traverses the carotid, ambient and choroidal cisterns which may supply adjacent eloquent structures including the optic nerve, medial temporal lobe and middle cerebral peduncle. (**B**) axial image of a maximum intensity projection of the same contrast enhanced MR angiogram which demonstrates the path of the AChoA in relation to the anterior and posterior circulation. (**C**–**E**) B1000 diffusion weighted axial images of the brain arranged caudal to cranial. These panels demonstrate restricted diffusion (hyperintensity) involving the AChoA territory in keeping with acute infarction.There is involvement of the hippocampus/mesiotemporal lobe (**C**), internal limb of the internal capsule encroaching on the lateral thalamus (**D**), and the corona radiate extending towards the caudate tail (**E**).

Within the clipping cohort, both patients had AChoA territory infarcts. The post-procedural mRS were 5 and 3, respectively (Figure 2).

#### 3.4.2. Recurrence and Retreatment

**Figure 3:**
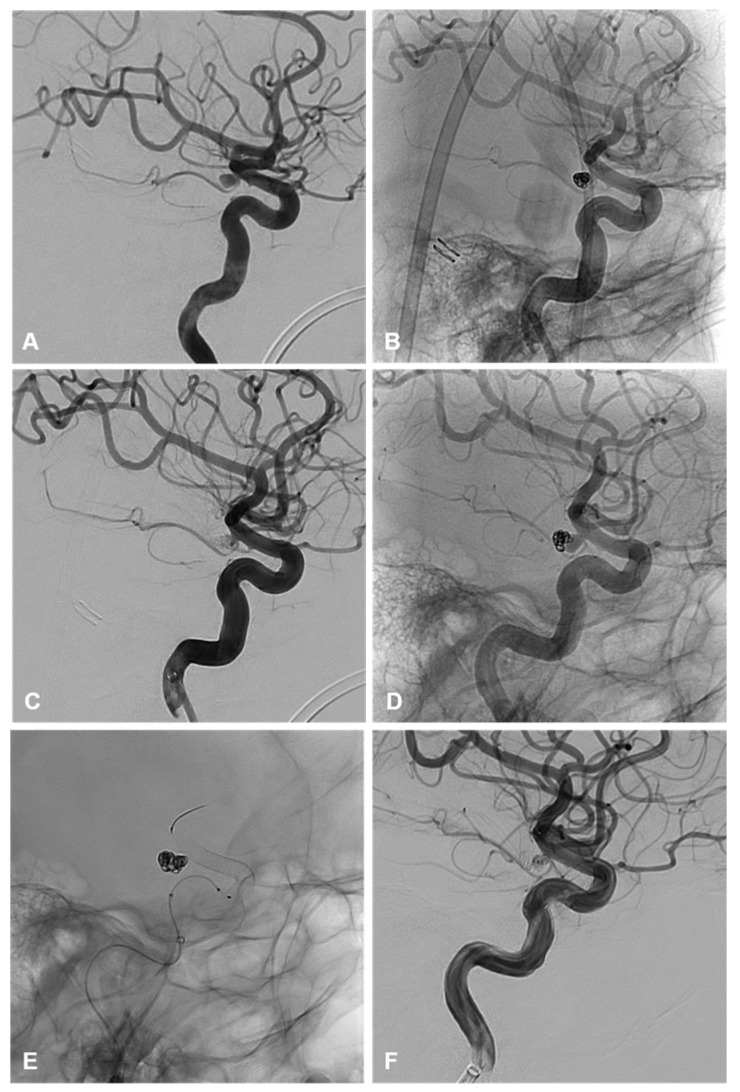
(**A**–**C**) demonstrate lateral angiographic images from the initial treatment of an unruptured AChoA aneurysm with primary coiling. (**A**) demonstrates a 4 mm smooth and saccular AChoA aneurysm that incorporates the origin of the AChoA at its neck. (**B**,**C**) are the unsubtracted and subtracted images of the final coil mass demonstrating a mRRC II and patency of the AChoA. (**D**–**F**) demonstrate the lateral angiographic images of the second treatment with coiling and flow diversion following recurrence. (**D**) is the unsubtracted image demonstrating coil compaction with an enlarged neck residuum prior to retreatment. (**E**) demonstrates the unsubtracted image of the final coil mass and flow diverter. (**F**) demonstrates the final subtracted angiographic run post re-treatment with coiling and flow diversion with preserved patency of the AChoA and some filling of the coil interstices. Follow up MRA at 3 months demonstrated complete occlusion of the aneurysm.

A total of 6/31 patients (19%) were retreated while 25/31 (81%) did not have a recurrence or need retreatment. Within the endovascular cohort, 3/24 (12.5%) patients required retreatment and 21/24 (87.5%) did not require any further treatment on follow-up (Table 3). Of those requiring retreatment, 2/3 (66%) were ruptured on initial treatment (Figure 1). One patient was initially treated with primary coiling and retreated by flow diverter stent, following recurrence after 5 months. The second patient was initially treated with primary coiling and was retreated with clipping due to recurrence after 3 months. Of these, 1/3 (33%) were unruptured on initial treatment with primary coiling and were retreated with a flow diverting stent (Figure 3).

Within the clipping cohort, 3/7 (43%) patients required endovascular retreatment and 4/7 (57%) patients did not require any further treatment on follow-up. Of those requiring retreatment, 2/3 (66%) were ruptured on initial treatment with clipping and required endovascular retreatment. One patient was an immediate post-clipping residuum requiring primary coiling and the second patient reoccurred after a year and required a combined balloon and stent-assisted coiling. The third patient requiring retreatment was unruptured on initial treatment with clipping and required endovascular retreatment with BAC and FDS. This recurrence occurred >10 years post-clipping and was identified on routine surveillance for additional unsecured intracranial aneurysms.

#### 3.4.3. Other Complications

In all treatment cases, there were no intra-procedural or post-treatment ruptures or re-ruptures on follow-up. Of these, 1/5 (20%) of the endovascular cases, requiring an intracranial stent or flow diverting stent, had in-stent stenosis on long-term follow-up that was asymptomatic and did not require an angioplasty or re-stenting. None of the treated patients died during hospital admission or due to clipping or endovascular treatment. No delayed surgical complications, such as surgical site infections, were reported.

### 3.5. Vasospasm

A total of 11/17 (65%) patients with a ruptured aneurysm had moderate or severe vasospasm; 9/11 (82%) had a modified fisher scale of 4 and 2/11 (18%) had a scale of 2.

### 3.6. Clinical Outcome

The clinical outcome for all patients was evaluated using the modified Rankin scale before and after intervention at the last available follow-up. Of the 19 patients who underwent primary endovascular treatment (13/19 ruptured), 1 case was lost to follow-up. In total, 14/18 (78%) patients had a good outcome with mRS ≤ 2. The median follow-up time was 32 months with an interquartile range of 50.5 months.

Of the 6 patients who underwent primary clipping (5/6 ruptured), 4/6 (67%) had a good outcome with mRS ≤ 2. The median follow-up time was 47 months, and the interquartile range was 141 months. With regards to the ruptured cohort, all patients had a preadmission mRS of 0. Within the endovascular cohort, 53% returned to baseline mRS 0 at follow up, 62% had a good outcome with an mRS ≤ 2, and 23% had a poor outcome of mRS > 2.

Within the clipping cohort, 50% returned to baseline mRS 0 at follow up and 50% had a poor outcome with an mRS > 2.

#### 3.6.1. Outcome in Patients with a Favorable Hunt and Hess Scale

Of the 19 patients who underwent primary endovascular treatment (1 lost to follow-up), 16/18 (89%) had a HH scale of 0–3. Of these, 12/16 (75%) had a favorable clinical outcome and 4/16 (25%) had a poor outcome. Of the 6 patients who underwent primary clipping, 5/6 (83%) had a HH scale of 0–3. Of these, 4/5 (80%) had a favorable outcome and 1/5 (20%) had a poor outcome (Table 4).

#### 3.6.2. Outcome in Patients with a Favorable WFNS

Of the 13 patients who underwent primary endovascular treatment for rupture, 1 patient was lost to follow-up. Of the 12 remaining patients, 10/12 (83%) had WFNS scale of I–III. Of these, 7/10 (70%) had a favorable clinical outcome and 3/10 (30%) had a poor outcome. Of the 4 patients who underwent clipping for rupture, 3/4 (75%) had WFNS scale of I–III. Of these, 2/3 (67%) had a favorable outcome and 1/3 (33%) had a poor outcome.

## 4. Discussion

In this section, different aspects of anterior choroidal artery aneurysm treatment, complications, and clinical outcomes were considered. This is an exploratory study with a limited sample size due to the low incidence of AChoA aneurysms. This naturally limits statistical power and precludes subgroup analysis. Allowing for this, we analyze and discuss our results to draw some meaningful conclusions for physicians when managing patients with AChoA aneurysms.

### 4.1. Anterior Choroidal Artery Anatomy

AChoA arises from the dorsal surface of the ICA (Internal carotid artery) in 98% of cases [4,6]. The cisternal segment traverses the carotid and ambient cisterns, where perforating arteries originate [7]. These perforators supply eloquent structures, including the optic tract, internal capsule (posterior limb, genu, and retrolenticular parts), globus pallidus, caudate tail, lateral thalamus, cerebral peduncle, hippocampus, and amygdala [8,9]. The choroidal segment traverses the choroidal fissure, entering the temporal horn to supply the choroid plexus and anastomose with the choroidal system (Figure 1).

Within our cohort, four procedural related infarcts were documented, all of which were ruptured presentations. These are outlined in Table 2 under Section 3.4.1 (Procedure related infarction). In each case, the origin of the AChoA was incorporated into the neck of the aneurysm, which appears to be a risk factor for both endovascular and surgical approaches. A recent meta-analysis reports a low ischemic complication rate for patients treated with flow diversion as this appears less likely to compromise the parent vessel [10]. In our series, we have not seen ischemic complications in our patients treated with flow diversion. However, this treatment was reserved for elective cases only.

### 4.2. Follow Up—Impact on Complications and Clinical Outcome

In our study, the median follow up for endovascular cases is 32 months, compared with 47 months for clipping. This is considered a relatively short/medium term follow up for both cohorts and has implications on interpreting complications, including recurrence. The limited follow up and the shorter follow up for endovascular treatment may underestimate the true incidence of recurrence and recurrence requiring retreatment.

### 4.3. Endovascular Treatment—Complications and Clinical Outcome

In our series, there were no mortalities associated with endovascular treatment. Mortality in endovascular treatment of these aneurysms is often low, with a previously reported 1% mortality rate [2].

An AChoA territory ischemic complication rate of 4% was noticed. We also noticed a 4% catheter related thrombo-embolic infarct that resulted in transient hemiparesis. Our results are comparable to previous studies that reported 4–6.5% AChoA related infarction [2,11,12,13] and 4–5.4% for other thromboembolic complications [2,3,5].

Patients with ischemic complications presented in the immediate post-procedure period between 0 and 4 h. Other studies reported similar experiences [2,3]. We suggest that close monitoring of neurovitals in this period will help identify the early onset of focal neurology and may prompt management such as GPIIbIIIa inhibitor in select cases.

Our series demonstrated a relatively low recurrence rate for endovascular treatment, with only 3/24 (12.5%) patients requiring retreatment, which is comparable with other studies [5,12,13,14]. Two of these patients were ruptured, and all patients were initially treated with primary coiling.

One unruptured aneurysm, with a size of 11 mm and neck size of 3.2 mm, underwent primary coiling with mRCC of II. This residuum enlarged after 4 years and was retreated electively with FDS. In medium to large size unruptured AChoA aneurysms, FDS may be considered initially [15,16,17].

In our series, 78% of endovascular treatments had a good outcome with mRS ≤ 2, which is comparable to other studies [3,10]. This comprises 83% good outcomes for unruptured cases and 75% good outcomes for ruptured cases. The median follow-up time was 32 months, with an interquartile range of 50.5 months.

Our findings corroborate the results of previous studies that demonstrate a favorable safety profile of endovascular approaches for AChoA aneurysm treatment [10,18,19].

### 4.4. Clipping–Complications and Clinical Outcome

In our series, there were no mortalities associated with clipping. An AChoA territory infarct of 29% is comparable to other studies [20,21,22]. A 43% retreatment rate was noticed. One recurrence case occurred after more than 10 years of follow-up.

On review of these cases, the mean neck measured 3.5 mm and the origin of the AChoA was incorporated in 66% of cases. These morphological features are thought to be potentially contributing factors to the high recurrence rate seen [18,23].

**Figure 4:**
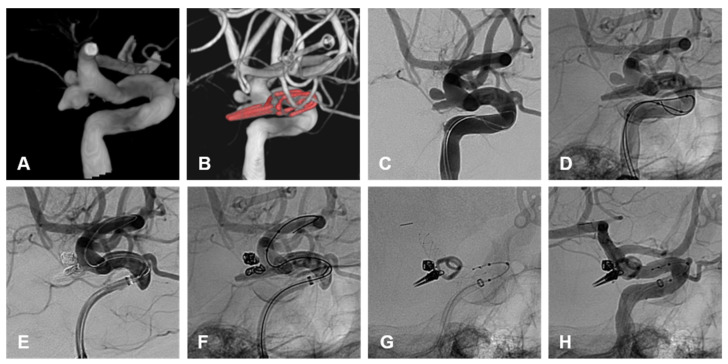
(**A**) demonstrates a 3D shaded surface display of a ruptured irregular AChoA aneurysm with a posteriorly pointing nipple thought to be the rupture point. Note is made of an additional unruptured aneurysm cranial to this. The patient underwent surgical clipping of the ruptured AChoA aneurysm and remodeling of the neck to preserve the AChoA origin. (**B**) The patient developed an enlarging residuum on routine surveillance imaging 52 months after initial treatment. This panel demonstrates a 3D shaded surface display of the clip partly obscuring the recurrence. Note is made of the location of both aneurysm necks, which are posterior to the ICA and not visualized adequately from a sub frontal approach. (**C**,**D**) are unsubtracted and subtracted lateral angiographic images demonstrating the recurrent AChoA aneurysm and the additional untreated and unruptured aneurysm cranial to this. The patient underwent elective re-treatment with balloon assisted coiling of both aneurysms, the results are seen on the subtracted and unsubtracted angiographic images (**E**,**F**). A braided stent was deployed within the ICA, across both aneurysm necks, to promote endothelialization. This is demonstrated on the unsubtracted final angiographic images (**G**,**H**).

All recurrences opted for endovascular retreatment with a variety of different endovascular approaches. None of these retreatments required further treatment on follow-up. This preference for endovascular retreatment suggests that the varied armamentarium of endovascular approaches makes it malleable to altered anatomy and possibly less favorable surgical conditions with AChoA aneurysm treatment. This includes the protective micro catheter technique, remodeling with balloons, and reconstructive techniques, using stents and flow diverters. These techniques would be suitable for primary and/or secondary treatment of these aneurysms [3,4,16] (Figure 4).

The demonstration of possible long-term recurrence may also warrant long-term follow-up for this cohort of patients [24]. 3D angiographic evaluation and mimicking the clipping approach allows for better planning and identification of the AChoA location in relationship to the aneurysm dome. This may be helpful, while dissecting the aneurysm neck, for pilot clip placement [25].

Surgical visualization of the aneurysm is often limited as it is within a deep and narrow space lateral to the ICA and in close relation to the skull base. The size and shape of the anterior and posterior clinoid processes, and the close relation to the uncus of the medial temporal lobe, may further complicate visualization, requiring extensive dissection. Retraction of the temporal lobe or uncus may result in rupture of the aneurysm or tearing the AChoA and its perforators. This is further complicated by the limited view of the aneurysm neck, AChoA origin, and its perforators, which are often obscured by the aneurysm and the ICA [4]. AChoA aneurysms lie in close proximity to the optic and oculomotor nerves, which are at risk of injury during dissection [26].

Temporary clipping, proximally of the ICA and distally of the A1 and M1, might be required to reduce the pressure within the aneurysm sac and reduce rupture risk of the inherently small and thin-walled aneurysm [6]. This requires a perforator and calcification-free zone for each clip to be placed effectively. These two factors do not complicate endovascular approaches. Excessive manipulation of the ICA during this process may result in rupture of the aneurysm.

Extensive dissection allows for better visualization of final clip placement and allowing for immediate clip repositioning or reconstruction if required [4,23]. In SAH, dissection of the subarachnoid space is more difficult due to acute swelling of the brain or adhesions from a previous SAH [27].

Ensuring that the AChoA territory is not compromised during temporary clipping and confirming patency of the AChoA at the end of the procedure are critical.

Intra-operative assessment using real time neurophysiological monitoring and qualitative measure of flow, with ICG video angiography, are used to help evaluate these [28,29].

Intraoperative neurophysiological monitoring, using MEP and SSEPs, is used to assess potential compromise of the AChoA territory during temporary clipping. Blood flow impairment to the internal capsule and cerebral peduncle, containing the corticospinal tracts, is monitored through MEPs caused by electrical stimulation, often of the hand motor cortex.

It was previously reported that changes in MEPs and SSEPs often lag behind clinical deficits, which may result in false negatives and a poor sensitivity of 33% [22]. In the same study, the use of ICG video angiography improved occlusion rates of aneurysms and clip repositioning, but there was no reduction in AChoA territory infarct. They also describe that 5/6 patients with AChoA territory infarct demonstrated a patent AChoA on immediate post-operative angiograms, suggesting that temporary flow arrest may lead to ischemia. The mean duration of temporary clip occlusion in their series was 5.6 min [22].

In our series, all patients underwent monitoring with MEPs and SSEPs. One of the two patients with AChoA territory procedure-related infarcts had compromised potentials in the upper and lower limbs. SSEPs returned to normal, but MEPs remained at 40% of baseline.

Despite the demanding procedural technicalities, this series demonstrates that a good clinical outcome (67%) with mRS ≤ 2 was achieved in the clipping cohort, which is comparable to other studies [14,20]

### 4.5. Limitations

This study has several limitations, including its retrospective nature, making it inherently prone to selection bias. The number of endovascular treatments is similar to other small case series but, in the grand scheme of things, represents a relatively small number, thereby affecting the power of the dataset. The small number of clipping-treated aneurysms precludes accurate comparisons between the two treatment arms. The study reviews the experience of a single center, and although there are multiple neurosurgical and endovascular operators, this may limit the generalization of these results. The absence of imaging core lab adjudication for obtaining aneurysm measurements and characteristics means that there is potential variability and bias. To help mitigate this, the measurements were blindly performed by a neuroradiologist twice. Any discrepancies were reviewed by an additional party. All ruptured AChoA that presented to the institution were investigated with catheter angiography and therefore would be captured in our cohort. There may be selection bias for patients with unruptured AChoA who may have only had CTA or MRA imaging and, therefore, are not represented in our study. A well-designed randomized control trial would be the most accurate way of evaluating the efficacy and safety profile of both endovascular and clipping techniques in the treatment of AChoA aneurysms.

## 5. Conclusions

Clinical outcomes of both clipping and endovascular treatment of AChoA aneurysms look to be comparable. In this study, endovascular treatment demonstrated low recurrence rates and procedure-related thromboembolic complications in keeping with the current literature. Endovascular treatment demonstrated lower recurrence and thromboembolic rates compared to clipping. Both approaches are viable, though they present distinct risks and advantages.

## Figures and Tables

**Table 1:** PHASES scores in the endovascular and clipping cohorts with pre-procedural hemorrhage status.

	Ruptured		Unruptured		Total	
PHASES	Endo	Clip	Endo	Clip	Endo	Clip
4	8	3	1	1	9	4
5	3	1	4	0	7	1
6	1	0	0	1	1	1
7	1	0	0	0	1	0
10	0	0	1	0	1	0

**Table 2:** Morphological and clinical details of the four cases that had a procedural related infarct, all of which were treated as acutely ruptured aneurysms.

#	Treatment	ASA Use	AChoA Involvement	Size (mm)	Neck (mm)	Width (mm)	ASPECT Ratio
1	Clip	No	Yes	4.5	3.7	4	1.22
2	Clip	No	Yes	5	2.2	5.9	2.27
3	Coil	No	Yes	2.5	2.4	1.6	1.04
4	BAC	Yes	Yes	3.8	2	2.6	1.90

ASA acetylsalicylic acid; ASPECT Ratio (Dome/Neck); BAC balloon assisted coiling.

**Table 3:** Morphological and clinical details of the six cases that had recurrence requiring retreatment.

#	Initial Bleed	Initial Treatment	Reccurent Treatment	AChoA Involvment	Size (mm)	Neck (mm)	Width (mm)	ASPECT Ratio
**1**	No	Clip	BAC and FDS	No	4.5	3.6	2.5	1.25
**2**	Yes	Clip	Coil	Yes	4.5	3.7	4	1.22
**3**	Yes	Clip	BAC and SAC	Yes	5.6	3.2	4.2	1.75
**4**	No	Coil	FDS	No	11	3.2	7.5	3.44
**5**	No	Coil	BAC and SAC	Yes	2.5	2.2	2.8	1.14
**6**	Yes	Coil	FDS	Yes	4.1	2.3	4	1.78

ASPECT Ratio (Dome/Neck); BAC balloon assisted coiling; FDS Flow diverting stent; SAC stent assisted coiling.

**Table 4:** Clinical outcome for the endovascular and clipping cohorts based on the presentation Hunter and Hess scale.

	HH 0		HH 1–3		HH 4–5		TOTAL	
MRS	Endo	Clip	Endo	Clip	Endo	Clip	Endo	Clip
0–2	5	2	7	2	2	0	14	4
>2	1	0	3	1	0	1	4	2

## Data Availability

Data is unavailable due to ethical and legal restrictions.
